# The role of exosomal noncoding RNAs in cancer

**DOI:** 10.1186/s12943-019-0984-4

**Published:** 2019-03-09

**Authors:** Yan Xie, Wei Dang, Siwei Zhang, Wenxing Yue, Li Yang, Xingyu Zhai, Qijia Yan, Jianhong Lu

**Affiliations:** 1 Department of Pathology, Xiangya Hospital, Central South University, Changsha, 410080 China; 2 Department of Microbiology, School of Basic Medical Science, Central South University, Changsha, 410078 China

**Keywords:** Exosomes, Cancer, Noncoding RNAs, Function

## Abstract

Extracellular vesicles (EVs) membranes enclose nanosized vesicles with a size range of 30–150 nm and are plentiful in our body in both physiological and pathological conditions. Exosomes, a type of EV, are important mediators of intracellular communication among tumor cells, immune cells, and stromal cells. They can shuttle bioactive molecules, such as proteins, lipids, RNA, and DNA; however, the precise function of EVs remains largely unknown. In recent years, tumor-associated cargo in exosomes has been a hot topic in research, especially with respect to noncoding RNAs (ncRNAs). Herein, we review the role of exosomal ncRNAs, including miRNAs and long noncoding RNAs, in tumor biological processes. Clinically, exosomal ncRNAs may eventually become novel biomarkers and therapeutic targets in cancer progression.

## Introduction

Extracellular vesicles are small cell-derived membranous structures containing various endogenous cargos, such as proteins, lipids, and genetic material [1]. EVs were first discovered in 1983 in sheep reticulocytes. Johnstone named these structures “exosomes” in 1987. Secretion of EVs was initially described as the cell’s way of ridding itself of nonfunctional metabolites [2]. Upon in-depth study of EVs, however, researchers found that these “unnecessary compounds” exchange components between cells and play a key role in cell communication, the immune system, tumor metastasis, and other important pathways.

**Fig. 1:**
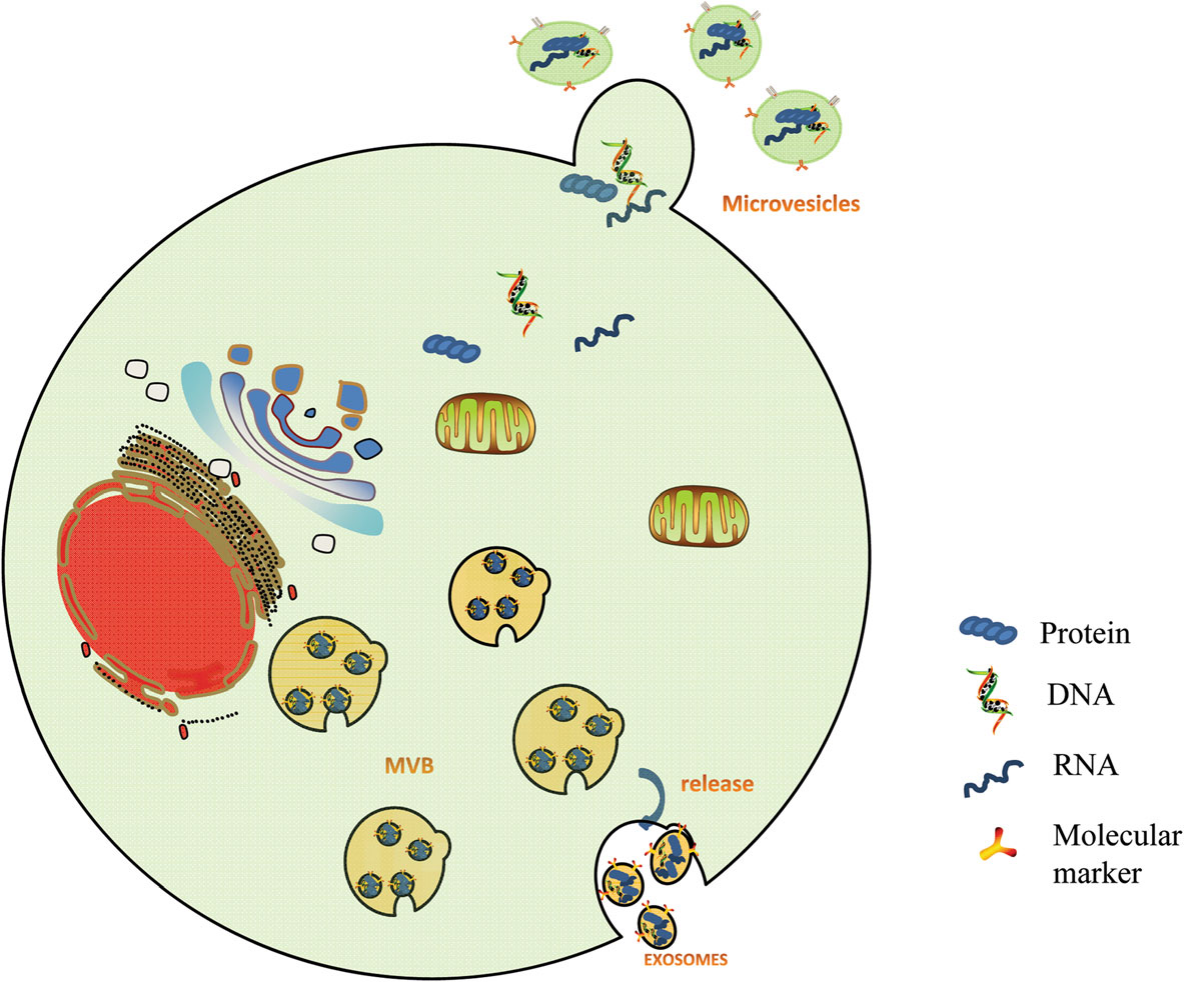
Biogenesis of exosomes and microvesicles. Multivesiculer bodies (MVBs) are derived from late endosomes, which are developed by early endosomes via endocytosis. Exosomes are released after MVBs fuse with the cellular membrane. Microvesicles (50–1000 nm in diameter) are budding from the plasma membrane

EVs can be divided into two main types: exosomes and microvesicles, based on their differential modes of biogenesis and size (Fig. 1). Exosomes are generated within the endosomal pathway and secreted from the fusion of MVBs, a type of endosome, that are released by exocytosis after fusion of multivesicular bodies with the plasma membrane. Subsequently, exosome refers specifically to membrane vesicles with a diameter of 30–100 nm. In contrast, microvesicles arise through the outward budding of the plasma membrane and are formed by a direct, outward budding and pinching event. Microvesicles range in size from 50 to 1000 nm in diameter [3–5].

There are many sources of diagnostic medium from which to isolate EVs, such as the blood, urine, breast milk, saliva, etc. [6]. As we elucidate additional mysteries about EVs, increasing studies are focusing on the function of their differential cellular components, which can facilitate tumor progression by promoting tumor cell growth, cell invasion, metastasis, angiogenesis, and even tumorigenesis [3]. mRNAs have three basic segments, the 5′-untranslated region (5′-UTR), the coding region (CR, which encodes protein), and the 3′-UTR. During past decades, studies have been devoted to uncovering the mechanisms of EVs in regulating tumor occurrence and development, as well as unearthing novel and highly promising biomarkers and therapeutic targets. Some findings declare that mRNA residing in EVs from the parent cell can be translated in the recipient cell to program relevant functions [7]. MicroRNAs (miRNAs) are endogenous, small RNAs of approximately 19–22 nucleotides in length that have numerous important regulatory roles within cells. The complex regulatory network of miRNAs not only regulates expression of multiple genes through a single miRNA, but the combination of several miRNAs can also finely regulate expression of a single specific gene [8–10]. Long noncoding RNAs (lncRNAs) are greater than 200 nucleotides in length and are usually divided into five categories: sense, antisense, bidirectional, intronic, and intergenic. LncRNAs are involved in X chromosome inactivation, genomic imprinting, chromatin modification, transcriptional activation, transcriptional interference, nuclear transport, and many other important regulatory processes [11, 12].

### Exosomal RNAs in epithelial–mesenchymal transition

In various cell types, epithelial-mesenchymal transformation (EMT) is closely related to tumor invasion and metastasis [13, 14]. Epithelial cells undergo a transient structural change after EMT whereby their polarity is lost, so that their contact with surrounding cells and matrix is reduced. EMT is characterized by acquisition of a mesenchymal phenotype due to the loss of expression of keratin filament and E-cadherin and gain of expression of Vimentin, fibronectin, N-cadherin, α-SMA, and various proteases [15, 16]. In this regard, EMT promotes infiltration and migration of tumor cells, allowing tumor cells to evade surveillance of apoptosis.

MiRNAs that are selectively enriched in exosomes involved in EMT were revealed by Elvira Donnarumma et.al. Different experiments have verified that three miRNAs (miRs − 21, −378e, and − 143) are increased in exosomes from cancer-associated fibroblasts (CAFs) and can shuttle into breast cancer cells to promote EMT [17]. Elisabetta Bigagli and colleagues found that miR-210 is significantly up regulated in exosomes secreted by primary colon tumors, hindering E-cadherin re-expression in nearby metastatic cells [18]. Moreover, Xiao et al. identified two miRNAs (miR-191 and let-7a) that play a role in melanoma cell-derived exosome-mediated EMT. Importantly, let-7a promotes invasive ability through expression of E-cadherin and inhibition of Vimentin expression through targeting LIB28B and HMGA2. Furthermore, the MAPK pathway is one of the important links in exosome-mediated EMT [19]. Interestingly, a select set of miRNAs are incorporated into exosomes involved in EMT, especially miR-23a and are significantly increased in TGF-β-treated mesenchymal phenotypic lung adenocarcinoma cells [20]. Primary urothelial bladder cancer (UBC) cells were identified to alter expression of EMT genes, including SNAI1, TWIST1, ZEB1, ZO1, MMP1, LAMB3, and LAMC2 via secretion of exosomic lncRNA HOTAIR. Employing shHOTAIR in two human bladder cancer cell lines revealed that expression of the master regulators of EMT (SNAI1) was significantly reduced [21].

Additionally, there are a number of EMT-related exosomal noncoding RNAs that have been identified in corresponding malignances, such as breast cancer, colon cancer, lung adenocarcinoma, and bladder cancer. These exosomal-RNAs ingested by recipient cells can promote expression of mesenchymal-like cell properties, as well as reducing epithelial-like cell characteristics. EMT makes it easier for tumor-derived exosomes to enter the circulation and reach distant sites to form a premetastatic microenvironment.

### Exosomal RNAs induce angiogenesis

Tumor cells are very clever and can form new blood vessels to obtain oxygen and nutrients, which are indispensable for tumor survival [22]. Tumor-derived exosomes that have been identified have shown many RNAs that favor angiogenic activity. The most plausible explanation for this phenomenon is that these RNAs are interacting with cytokines, chemokines, and growth factors [23].

Hypoxia resulting from the instability of the tumor-associated microvasculature is a characteristic feature of malignant tumors because tumor cells can migrate to better nourished environments in distant organs. Malignant tumors under hypoxic conditions secrete many exosomes containing proangiogenic RNAs, which are associated with poor prognosis. MicroRNA-135b, derived from hypoxia-resistant MM (HR-MM) cells, suppresses expression of its target, factor-inhibiting hypoxia inducible factor 1 (FIH-1), in endothelial cells, thus fostering angiogenesis [24]. Of note, miR-210 is enriched in hypoxic exosomes from breast cancer cell, and the possible mechanism for this is that exosomal miR-210 may promote hypoxic signaling, such as through the HIF oxygen sensing pathway [25]. Vascular endothelial growth factor (VEGF) is an important mediator of inflammation and angiogenesis. Interestingly, a positive correlation exists between miR-21-derived from cigarette smoke extract (CSE)-transformed human bronchia epithelial (HBE) cells and increased expression of VEGF, which may contribute to angiogenesis. Moreover, activation of STAT3 is related to the cross-talk between these processes [26]. Min Gong et al. confirmed that exosomes secreted from mesenchymal stem cells (MSCs), including pro-angiogenesis microRNAs (miR-30b, 30c, 424, and let-7f), can upregulate the expression of pro-angiogenic factors [27].

In conclusion, many studies have demonstrated that RNAs play an important role in angiogenesis via secreting extracellular vesicles and promoting cell-to-cell communication.

### Exosomal RNAs stimulate a premetastatic niche and occurrence of metastasis

Exosomes play a unique role in multiple steps of the formation of the premetastatic niche (PMN) before metastasis. There are six characteristics of the premetastatic microenvironment defined by Yang Liu, including immunosuppression, inflammation, angiogenesis/vascular permeability, lymphangiogenesis, organtropism, and reprogramming, emphasizing the importance role of the premetastatic niche in tumor progression [28, 29].

Cristina Grange and colleagues demonstrated that MVs derived from CD105-positive human renal cancer stem cells were enriched in a set of mRNAs and microRNAs that molecularly characterize the lung premetastatic niche. They analyzed up regulated miRNA target genes predicted by the TargetScan algorithm and found that miR-29a, miR-650, and miR-151 were associated with tumor invasion and metastasis. MicroRNAs that stimulate growth factors are also found packaged in CD105^+^MVs, for example, VEGF, fibroblast growth factors 2 (FGF2), angiopoietin1, and ephrin A3, as well as MMP2 and MMP9. These factors contribute to generation of the lung premetastatic niche [30]. Additionally, a number of noncoding transcripts in tumor-derived exosomes have been identified by RNA sequencing, most of which are lncRNAs. Furthermore, tumor exoRNAs can activate lung epithelial cell TLR3 to recruit neutrophils due to the expression of chemokine receptors (CXCR1, CXCR2, CXCR4, andCCR2) being higher in tumor-bearing Tlr3^−/−^ mice, consequently inducing lung premetastatic niche formation [31]. Makiko Onoet et al. extracted exosomes from BM-MSCs and found that the breast cancer stem cell (CSC) marker CD44 was decreased when exosomes were cocultured with BM2 cells. Using qRT-PCR, miR-23b was enriched in those exosomes and was responsible for breast cancer cell dormancy in the bone marrow premetastatic niche by binding to the 3′ untranslated region (3’UTR) of MARCK5 [32]. Besides possessing the capability to promote metastasis in premetastatic niche directly, breast-cancer-secreted miR-122 down regulates the glycolytic enzyme pyruvate kinase, which facilitates disease progression. In this characteristic mechanism, high levels of miR-122 in EVs significantly decreased glucose uptake and lactate production in recipient cells owing to decreased expression of PKM2 and GLUT1 [33]. It is well known that bone metastasis usually occurs in patients with lung cancer, resulting in systemic spread of cancer cells. Hence, Zhen Xu and team-workers focused on recent research hotspots, including exosomes, up regulated miR-21 in solid tumors, and osteolytic bone metastasis. They identified that A549 cell exosome-derived miR-21 facilitates bone marrow monocyte (BMM) cell proliferation through targeting the PDCD4 pathway [34, 35]. In particular, the release of exosomal ncRNAs may play a role in the dynamic and reciprocal cross-talk between tumor cells and the metastatic niche [36]. In the case of brain metastasis, exosomal miR-19a carried by astrocytes is delivered to tumor cells by targeting the suppressor gene PTEN. PTEN-loss causes the activation of NF-κB and increased the expression of CCL2, thereby allowing tumor cells to extravasate to the brain [37]. The role of exosomal ncRNAs in microenvironment regulation is also a potential problem in cancer therapy.

In summary, different tumor types undergo directional premetastatic niche formation in different organs. Increasing studies have shown that RNAs in extracellular vesicles are involved in premetastatic niche formation, regulating tumorigenesis and development through a variety of different mechanisms.

### Exosomal RNAs in the immune response

The role of exosomes in immune response during tumor development depends on whether they are derived from tumors or from immune cells. Increasing studies have shown that there is a strong link between EVs and immune regulation, either through immune activation or immune suppression.

Using miRNA chip microarray analysis, Shu-biao Ye et.al. discovered five over expressed miRNAs (hsa-miR-24-3p, hsa-miR-891a, hsa-miR-106a-5p, hsa-miR-20a-5p, and hsa-miR-1908) in exosomes from patient serum and NPC cells. These tumor exosomes (TEX) can mediate T-cell dysfunction, for instance, by impeding differentiation of Th1 and Th17 cells while promoting Tregs. TEX can produce added pro-inflammatory cytokines via altering cytokine profiles. The underlying mechanism for this may be related to these miRNAs targeting elements of the mitogen-activated protein kinase (MAPK) signaling pathway to alter ERK and STAT protein phosphorylation [38]. Actually, pancreatic cancer (PC) cell-derived exosomes switch the differentiation of macrophages to the M2 phenotype, promoting immunosuppression and metastasis independent of HIF-1a or HIF-2a. Conversely, hypoxic exosomes were significantly reduced, as was expression of M2 markers on macrophages when using CRISPR/Cas9 to knock out miR-301a-3p in PANC-1 cells. PC-derived exosomes activated the PI3Kγ pathway to promote immunosuppressive-related genes in M2 macrophages [39]. Another study validated that two endogenous miRNAs, miR-142 and miR-223, expressed in macrophages have an intimate role in inhibiting HCC proliferation. Intercellular contact via exosomes is one of their functional pathways, and use of several inhibitors of gap junctions to disrupt actin microfilaments of the cytoskeleton demonstrated that the efficiency of miRNA transfer between human macrophages and HuH7 depends on intact connections between cells [40–42].

Whether they are secreted from immune cells or tumor cells, exosomes contribute to our understanding of the tumor microenvironment, their patterns of spread, and the type of immune responses they can cause. Different types of exo-RNAs can regulate the progression of different tumors through differential signaling pathways. When further utilizing these exo-RNAs as therapeutic targets, we can identify exact disease etiology and prescribe medicine for a patient according to their illness.

### Exosomal RNAs are involved in therapeutic resistance

Extracellular vesicles may spread resistance capacity between heterogeneous populations of tumor cells, and ultimately blocking the successful treatment of many cancers [43]. Mechanisms of how tumor cells convey a beneficial environment for their growth, metastasis and therapy resistance are vast.

The results from Peiming Zheng demonstrated that exosomes derived from tumor-associated macrophages (TAMs) that secreted microRNA-21 (miR-21) promoted cisplatin (DDP) resistance in gastric cancer cells. Using Cy3-labeled miR-21, they demonstrated that gastric cancer cells can ingest M2-exosomes and play a role in the development of resistance. Elevated miR-21 levels effectively reduce apoptosis and chemosensitivity by decreasing PTEN expression, while increasing phosphorylation, resulting in enhanced cisplatin resistance in GC patients [44, 45]. Furthermore, Kishore B. Challagundla et al. focused on exosomic miR-21 and miR-155 due to their close association with inflammatory responses in the tumor microenviroment. As expected, high miR-155 expression in neuroblastoma (NBL) cells is caused by secretion of exosomic miR-21 when human monocytes were cocultured with NBL cells. The function of miR-155 is to specifically inhibit expression of TEPF1, thereby enhancing the activity of telomerase, which in turn triggers the CDDP resistance phenotype [46, 47]. Docetaxel and adriamycin resistance have been studied in breast cancer, and Wei-xian Chen et al. used different drug densities to examine the chemoresponse of MCF-7/Adr and MCF-7/Doc, which are the resistance variants of MCF-7/S, identifying three miRNAs (miR-100, miR-222 and miR-30a) that were enriched in both A/exo and D/exo [48]. CSCs, which have the ability of “self-renewal” and “differentiation”, are also involved in therapy resistance. In colorectal cancer (CRC), carcinoma-associated fibroblasts derived exosomes can increase the number of CSCs and trigger chemoresistance by activating WNT signaling pathway [49]. Conversely, Guohua Lou et al. demonstrated that AMSC-derived exosomes containing miR-122 can enhance the chemosensitivity of hepatocellular carcinoma (HCC) [50].

Emerging evidences have shown the potential mechanism of ncRNAs in regulating cancer progression. Transcription factors (TFs) regulates the expression of ncRNA precursors, which may in turn regulate TFs. MYC is a crucial TF that is highly expressed in a lot of human cancers. Both miRNA and lncRNA can directly affect the stability, transcription level and activity of MYC [51]. MiRNAs negatively regulate the expression of a target gene by reducing the stability of the target mRNA or post-transcriptionally inhibiting the translation process. A lncRNA can act as a competitive endogenous RNA (ceRNA) by competing with other RNA transcripts for the same miRNA [36, 52].

**Fig. 2:**
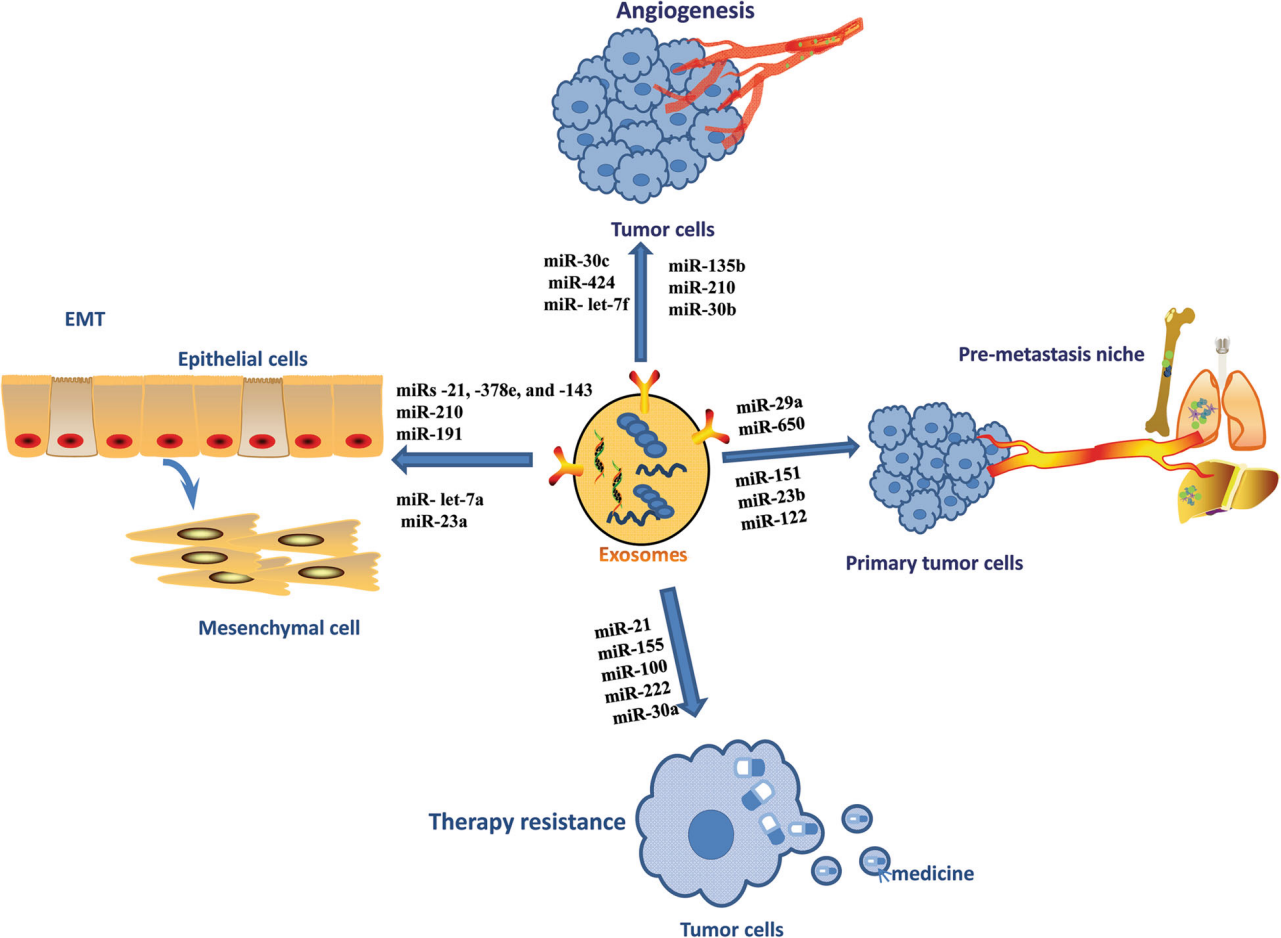
Summary of exosomal ncRNAs mediated functions. Exosomes contain lipids, proteins and nucleic acids. Exosomal noncoding RNAs, especially miRNAs, can regulate tumor progression, such as EMT, angiogenesis, pre-metastasis niche formation and therapy resistance

When these exosome-loaded miRNAs transfer to sensitive cells, the cell cycle and apoptosis pathways are changed to protect tumor cells from drug therapy. To our knowledge, investigating the mechanisms of resistance of exosomal RNAs with respect to therapeutic resistance could improve treatment options (Fig. 2).

### Potential roles of exosomal RNAs in cancer diagnostics and therapy

#### Exosomal ncRNAs as cancer diagnostic biomarkers

Exosomes, whether from tumor or normal cells, have emerged as ideal biomarkers due to their inherent properties. Increasing academic works have implicated exosome-delivered miRNAs as playing a role in cancer diagnosis and prognosis, as well as for prediction and monitoring of anticancer therapies [53–56]. A variety of cells release exosomes in body fluids, such as the blood, urine, saliva, breast milk, and ascites [57–61]. Because of convenient collection methods, they offer a key advantage to serve as a liquid tool for noninvasive clinical testing.

In a report by Corinna Eichelser et al., it was demonstrated that levels of exosomal miR-373 were specifically increased and higher in triple-negative breast cancer patients, highlighting the potential role of miR-373 as a plasma-based biomarker of more aggressive tumors [62]. Exosomal miR-105 has been associated with the premetastatic stage in breast cancer patients. Expression profiling technology (microRNA expression profiles) has been applied to determine the RNA component in EpCAM-positive exosomes, which were isolated from women with benign and malignant ovarian disease [63]. Despite these exo-miRs failing to identify all stages of ovarian cancer, eight tumor-specific miRNAs can distinguish malignant cancer from benign disease. In another study, Noah I. Hornick et al. developed a xenograft model of AML disease and corroborated that several miRNAs in serum exosomes have significantly different expression levels in leukemia-engrafted mice. Furthermore, evaluating three AML patients and equal number of nontumor patients revealed that in circulating patient exosomes, these miRNAs were markedly higher in expression compared to controls [64]. In prostate cancer (PC), exosomal miR-141 and miR-375 are remarkably stable miRNA forms in serum that can better discriminate metastatic PC patients from healthy individuals with significant specificity and sensitivity [65, 66]. Another article published in J Cancer Res Clin Oncol demonstrated that long noncoding RNA ZFAS1 was elevated in GC patient’s serum exosomes, indicating that lncRNA ZFAS1 may paly active role in GC progression. In this study, exosomal lncRNA ZFAS1 may represent a better biomarker for GC diagnosis [67]. Exosomes contribute to cancer progression through enhancing neighboring and distant cells’ communication. Measurement of these miRNAs may provide novel clinical utility based on body fluid detection (Table 1).

**Table 1:** Clinical application of exosomal RNAs as biomarkers

Cancer type	ncRNA	Exosome source	Cancer outcome	Application	Purification strategy	Reference
Breast cancer	miR-105	Serum	Enhance cancer progression and metastasis. Poor prognosis	Early diagnosis of BC metastasis	Ultracentrifugation	[29]
Esophageal Cancer	miR-93-5p	the Blood (Serum/Plasma)	Promote cancer cells’ Proliferation. Poor prognosis	Early diagnosis	Differential centrifugation and ultracentrifugation	[55]
Gastric cancer	LINC00152	Plasma samples	Not mentioned	Early diagnosis	Total Exosome Isolation Reagent	[54]
Colorectal cancer	lncRNA-GAS5	Serum	Inhibit CRC cell proliferation, migration and invasion. Favorable prognosis	Diagnosis and prognosis	Differential ultracentrifugation	[53]
Triple-negative breast cancer	microRNA-373	Serum	Inhibit cancer cells’ apoptosis. Poor prognosis	Early diagnosis	ExoQuick	[62]
Ovarian cancer	miR-21, miR-141, miR-200a, miR-200c, miR-200b, miR-203, miR-205 miR-214	Sera specimens	Poor prognosis	Early diagnosis	A modified magnetic activated cell sorting (MACS) procedure	[63]
Acute Myeloid Leukemia (AML)	let-7a, miR-99b, −146a, −155, −191, − 1246	Serum	Poor prognosis	Therapy monitoring	Differential centrifugation and ExoQuick	[64]
Prostate cancer (PC)	miR-141 miR-375	Serum, Plasma and Urine	Promote metastasis Poor prognosis	Early diagnosis	A filter concentrator with a 150-kDa molecular weight cutoff	[65]
Gastric cancer	LncRNA- ZFAS1	Serum or Sera	Enhance GC cell proliferation and migration Poor prognosis	Early diagnosis	Ultracentrifugation	[67]

Note: all the ncRNAs as biomarker are upregulated in the related cancers

#### Applications of exosomal ncRNAs in cancer therapy

It is important to consider that the existence exosome cargo might lead to a novel nanobiomedical therapeutic approach for cancer [68, 69]. For instance, it is worthy of further investigation that depleting EVs or blocking EV uptake pathways could be used to cure cancer patients [70]. Aethlon Medical has developed a novel device strategy, the Aethlon ADAPT™ (adaptive dialysis-like affinity platform technology) system, that can gather blood components < 200 nm that interact with the device’s immobilized affinity agents, resulting in cleaning tumor-derived exosomes and other oncological components [71]. Tullis et al. conducted the first ADAPT™ device study to treated hepatitis C virus (HCV) patients by capturing viruses with the lectin *Galanthus nivalis* agglutinin (GNA) of the affinity matrix [72]. Therefore, anti-HER2 antibodies could be used on the device to remove breast cancer-derived EVs, especially in HER2-overexpressing breast cancer.

Moreover, as endogenous nanocarriers, exosomes can be therapeutically targeted to deliver anti-cancer cargos to malignant cells [73]. This emerging novel therapeutic approach has some advantages, such as harboring a high payload of drugs, multiple drug loading, protecting contents from drug degradation, enhancement of endocytosis, lack of toxicity, and target specificity [74]. In Shin-ichiro Ohno and colleagues’ research, they introduced let-7 miRNA into GE11-positive exosomes and injected exosomes into RAG2−/− mice with EGFR-expressing xenograft tumors. Let-7a–containing GE11-positive exosomes released therapeutic molecules to suppress tumor growth [75]. A rat model by Mark Katakowski et al. utilized ‘self’ marrow stromal cell (MSC) exosomes. Transfecting a miR-14b expression plasmid into MSCs followed by intratumor injection of harvested exosomes resulted in significantly inhibited EGFP expression and reduced tumor volume in a glioma xenograft model [76, 77]. Even so, there are many uncertain factors that could influence the therapeutic potential of exo-miRNAs, and the effect of exosome-mimetic nanovesicles versus exosomes warrants further study.

Recently, one of the most forward therapeutic approaches developed monopolized on the fact that exosomes can be considered vaccines to stimulate antitumor immunity [78]. Several phase I clinical trials are devoted to exploring the clinical efficacy of dendritic cell-derived exosomes (DC-Exos). Bernard Escudier et al. used DC-Exos pulsed with MAGE3 peptides to treat stage III/IV melanoma patients. DC-Exos exhibited the ability elicit T cell immune response and enhanced NK cell function in a dose-dependent manner [79]. In another clinical trial, exosomes from the ascites of colorectal cancer (CRC) patients in combination with granulocyte-macrophage colony-stimulating factor (GM-CSF), rather than ascite-derived exosomes (Aex) alone, induced antigen-specific T-cell response [80]. Nonsmall cell lung cancer (NSCLC) treated with DCs-Exos led to 30% of patients developing DTH reactivity against MAGE peptide and increased NK lytic activity in 50% of patients [81]. Exo-targeted tumor cell vaccines may stimulate both innate and adaptive immunity. In this regard, DC-derived Michael A Morse Exos-based vaccines may exploit a new highly effective strategy against tumors. Sushrut Kamerkar et al. have illuminated that exosomes can be utilized to facilitate therapeutic targeting of oncogenic KRAS in pancreatic cancer. They injected siRNAs into the exosomes by electroporation, and found that CD47+ exosomes extends its life in the circulation by releasing ‘don’t eat me’ signals, making targeted therapy more effective [82]. Analogously, Haiyang Zhang et al. revealed that exosomes coated with si-HGF-1 significantly reduced the expression of HGF and VEGF, and thus effectively preventing gastric cancer from worsening [83]. In some ways, although these related cancer treatments have only been implemented in basic medical and phase I clinical aspects, these studies have brought new hope to cancer treatment.

### Isolation and collection of exosomes

Since exosomal ncRNAs have great potential of application, the isolation of exosomes becomes a technical concern. There several methods currently reliable for isolating exosomes from biopsy liquids. First of all, various commercial exosome extraction kits have appeared. The reagents are combined with water molecules, the less soluble exosomes can be precipitated by a centrifugal speed of approximately 1500 g [84]. Ultracentrifugation is another method to isolate exosmoes according to the density and size of the particulate constituents in biopsy liquids [85, 86]. Differential ultracentrifugation is one way usually used, containing several centrifugation procedures with different speed and duration to exclude cells and cell debris [87]. Ultrafiltration membranes are also popular for selectively separating exosomes with different molecular weight cut-off (MWCO) [88]. Another size-based separation technique is called as size exclusion chromatography (SEC), in which a porous multichannel stationary phase is utilized to make up of hetero-porous cross-linked polymeric gels. This is a kind of efficient and specific technique based on antibody-ligand interaction by capturing exosomal membrane’s specific surface markers for the first step [89–91]. Moreover, other effective techniques are developing for exosomes enrichment, including alternating current electrokinetic (ACE) microarray chip device and acoustic wave screening device based on new acoustic fluid technology [92, 93]. On the other hand, exosomes can be used for drug delivery in cancer therapy. To achieve this goal, a large number of drug-loaded or beneficial ncRNA-loaded exosomes may be needed. Senthilkumar Kalimuthu and his colleagues cultured mesenchymal stem cells (MSCs) in exosomes-depleted FBS containing different concentrations of Paclitaxe (PTX). When PTX is absorbed by the cells, it is encapsulated into the exosomes. Therefore, they can obtain a large amount of PTX-MSC-EXOs by ultracentrifugation for the purpose of breast cancer treatment [94]. Similarly, doxorubicin-loaded exosomes are prepared in vitro by incubating U937 cells with doxorubin. The artificial doxorubicin-loaded nanocapsule can be used to target colorectal cancer [95]. Bernard Escudier et al. separate monocyte derived-DC (MD-DC) from whole blood of patients. After proliferating in primary culture for 5 days, MD-DC was incubated with MHC/peptide complexes to extract exosomes. Quality-controlled exosomes were then reintroduced into the patient’s body to treat melanoma [80].

## Conclusion and future perspectives

Increasing evidence concerning EVs, in particular exosomal ncRNAs, have revealed their important roles in cancer development and potential application over the past few years. EV-derived ncRNAS are involved in oncogenic transfer, angiogenesis, immune modulation and premetastatic niche formation (Fig. 2).

Tumor-originated exosomes contain numerous RNAs that can inhibit the occurrence and progression of tumors. The most important EV components are microRNAs and lncRNAs. These types of RNAs dysregulate the expression of molecules associated with EMT and angiogenesis by cooperating with related signaling pathways in recipient cells. Moreover, these packaged “harmful” RNAs can also disrupt the microenvironment in secondary organs and tissue sites, promoting nonmalignant tissues to exhibit increased tumorigenic heterogeneity and promoting acquisition of a premetastatic niche. On the other hand, immune cell-derived exosomal RNAs play a role in the immune response. They have modest effects on the composition and activation of T cells, B cells, and other immune cells. With the complicated immunomodulatory effects, there will be a fierce confrontation between tumor cells and immune cells, but tumor cells eventually acquire therapeutic resistance. Exosomes are key substances that maintain the homeostasis of non-CSCs and CSCs. So far, we have not yet fully understood how non-coding RNAs in different CSC-derived exosomes play in tumors. They are like double-edged swords, in-depth research will pave the way for accurate and effective therapy, which depends on the exosomal characteristic. The biggest challenge for researchers and doctors is undoubtedly therapeutic resistance appearing during cancer treatment.

It is noteworthy that a variety of exosomes constituents provide emerging diagnostic methods and treatments for tumors. Clearly, the special RNA contained in corresponding exosomes is one of the most robust surrogates of tumors and reflects the current state of the tumor. Therefore, we can design targeted drugs for these RNAs and molecules that are closely related to these RNAs, furthering personalized medicine. Apart from the research progress in mechanism, a further challenge in clinical application on which we need focus is the “harmful” RNAs in exosomes and how to control the dosage of medicine to be used in exosomal therapy. Another unexplored problem in the field is how to guarantee the safety and quality of new methods for the isolation and utilization of exosomes.

With progressively better understanding of the nature of exosomes, corresponding diagnostic and therapeutic techniques are also improving. Future studies will likely put more energy into in vivo models and clinical application in order to help elucidate these questions.

## Abbreviations

3′-UTR: The 3′-untranslated region
5’UTR: The 5′-untranslated region
Aethlon ADAPT™: Adaptive dialysis-like affinity platform technology
AMSCs: Adipose tissue-derived MSCs
BMMs: Bone marrow monocyte cells
CR: The coding region
CRC: Colorectal cancer
CSC: Cancer stem cells
CSE: Cigarette smoke extract
DC-Exos: Dendritic cell-derived exosomes
DDP: Promoted cisplatin
EMT: Epithelial-mesenchymal transformation
EVs: Extracellular vesicles
FGF2: Fibroblast growth factors 2
FIH-1: Factor-inhibiting hypoxia inducible factor 1
GM-CSF: Granulocyte-macrophage colony-stimulating factor
GNA: Galanthus nivalis agglutinin
HBE: Transformed human bronchia epithelial
HCV: Hepatitis C virus
HR-MM: Hypoxia-resistant MM
lncRNAs: Long non-coding RNAs
MAPK: Mitogen-activated protein kinase
miRNA: MicroRNA
MSC: Marrow stromal cell
MSCs: Mesenchymal stem cells
NBL: Neuroblastoma
ncRNAs: Non-coding RNAs
NSCLC: Non-small cell lung cancer
PC: Pancreatic cancer
PC: Prostate cancer
PMN: Pre-metastatic Niche
TAMs: Tumor-associated macrophages
TEX: Tumor exosomes
UBC: Urothelial bladder cancer
VEGF: Vascular endothelial growth factor
